# Thoughts

**Published:** 1889-11-23

**Authors:** 


### Words of Consolation.

### THOUGHTS.

(*For Reading to the Sick.*)

What are you thinking about? Your pleasures or your pains? How you enjoyed yourself in the past, how jolly and gay you mean to be in the future? Or are you looking at the dark side of the picture, and can recall nothing but your trials, your aches and pains, and are anticipating the worries in store for you? Whichever view you take of life depends in some measure on the temper you were born with, but still more on the way you guide your thoughts. If you naturally hate to see sad sights, or hear of sad things, or even to dwell on serious things such as death, and Heaven, and hell, you will be always trying to drive away the remembrance of each and all of them by amusing yourself; you will like to eat, drink, and be merry, forgetting, with the rich man in the parable, that perhaps "this night thy soul will be required of thee." In a business-like point of view, is it not better to be prepared for all unexpected events?

Do you remember the story of the young man who on coming into his property at an early age, began to lay plans for enjoying his life. "First," he said, "I shall go to balls and theatres and all sorts of amusements, and see life." "And when you are tired of that," said the old friend to whom he was talking, "what will you do?" "I shall have horses and dogs, and hunt and shoot," was the reply. "And what next?" said the old gentleman. "I shall travel and see different countries." "And then?" continued his old friend. "I shall marry and settle down," said he. "And then?" "I shall probably have a family, and perhaps become a magistrate." "And then?" "Oh then I suppose I must die." "And then?" But the young man had no answer to such a question; he had not thought about what was to be his fate in the next world. Don't you think it would have been better for him if he could have made a reply? And about yourself, have you given any thoughts to your latter end? Now you are on a sick bed will it not be wise just to bring it to your mind? Yes, it really is true wisdom to be sure of what is in store for you. Just suppose that somebody you loved and trusted told you he had a home ready for you whenever you were prepared for it, but it was such a pure, beautiful, clean place that you could take none of your old clothes or furniture with you, and you must have white, clean garments, and gold ornaments, and precious stones to adorn you. I think you would try immediately to picture to yourself what it was like, and how you could get rid of your old clothes and everything that was ugly and vile, and if your kind friend gave you a book which contained every instruction on the subject, I believe you would be always studying this book and getting by heart what was written in it. Well, God has made you such a promise and given you in the Bible rules to guide you in the path to the heavenly home, and receipts for making your garments clean and white. To keep in the first you must ask your Heavenly Father to give His angels charge over you to keep you in all His ways; to do the latter you must wash yourself and them in the blood of the Lamb, our Lord Jesus Christ, who, by His prophets, tells us, "Though your sins be as scarlet they shall be white as snow." The filthy rags of wickedness and sin must be thrown away, the hindrances of wealth or business, those things which furnish our lives, must be left behind, and the gold and precious stones of love and righteousness and good works for God's glory must be sought. A woman may add the ornament of a meek and quiet spirit. When the Blessed Virgin, our Lord's mother, was told of all the wonderful things that would happen to her, she pondered them—that is, thought of them in her heart—and we are told in the Psalms, "Whosoever is wise will ponder these things, and he shall understand the loving kindness of the Lord."

